# Supplementary material for: Identification of a novel path for cerebrospinal fluid (CSF) drainage of the human brain
Source: PLoS One. 2023 May 4;18(5):e0285269. doi: 10.1371/journal.pone.0285269 (PMC10159342; doi:10.1371/journal.pone.0285269)
Supplement: Attachment — Submitted filename: Response to Reviewers.doc [file pone.0285269.s001.doc]

Response to Reviewers,

Alvan Ukachukwu, MD, MSc.GH
Academic Editor
PLOS ONE

1. The paper was re-written according to the PLOS ONE guidelines and formatting.
2. This study was reviewed by the IRB committee and it was determined that it did not require oversight or approval. This is stated in Materials and Methods.
3. The identification of CSF channels in the sagittal sinus is the focus of this work and the main part of the study, and this has not been published in a peer-reviewed journal. In addition, we have not described the intracranial CSF system. The prior work on the CSF drainage of the neck was verified in this paper with new dissections, and only included to complete the circuit from sagittal sinus to subclavian vein. We re-wrote the paper to emphasize the new features and to avoid “dual publication”.
4. We changed the title to simplify the message for a general audience. The identification of CSF channels in the sagittal sinus is a novel findingto the best of our knowledge We performed our historical review at Countway Medical Library Historical Books Collection.
5. The manuscript was amended to include the abstract after the Title Page.

Thank you for allowing us to re-submit a major revision. The paper was completely re-written to address the above comments and to hopefully make it interesting to the general scientific and medical community.

Response to Additional Editor Comments:

1. Medical illustrations with coronal and sagittal views were included to show the new model of CSF drainage as Figs 9a and 9b.
2. Medical illustrations with coronal and sagittal views were included in the INTRODUCTION to show the accepted model of CSF drainage as Figs 1a and 1b.
3. We included our confocal imaging of LYVE-1 as the lymphatic marker, and CD105 for our vascular endothelial cell marker. Podoplanin was no different from LYVE-1, so was not included to be concise. The positive vimentin image was included. These are Figs 7a-c. We re-wrote the paper to focus on the gross anatomy, and avoided making definite claims that CSF channels are not lymphatics. We are aware of Loveau’s and others’ work on meningeal lymphatics, and want to avoid this debate and focus on the identification of new anatomy in the sagittal sinus.
4. We discussed the effects of posture on CSF drainage and included reference 30. However, if possible, we would like to include a brief discussion of spaceflight-associated neuro-ocular syndrome because of the similar effects of microgravity and the supine position on CSF volume (both increase cerebral ventricular CSF volume). We included references 27-29 and 31 to clarify this topic.

Thank you again for allowing us to revise this manuscript.

Reviewer #1:

1. It's very difficult to interpret what is going on from the images. The authors should include an anatomic drawing that guides the reader to what will be seen in the images. Perhaps a diagram could also be included that outlines the understand of CSF drainage before this finding and how that understanding should change based on this new info. **RESPONSE**: We included medical illustrations of the accepted model of CSF drainage (Figs 1a and 1b) and the amended model of CSF drainage (Figs 9a and 9b) based on our findings.
2. Before jumping to conclusions about weightlessness, the authors might want to think about what this means for postural changes. The canalicular system may be gravity dependent but so are the veins and arteries. What are the implications of this for the upright vs. supine posture? **RESPONSE**: We included a discussion of how posture affects CSF volume with citation of reference 30. We also briefly discussed spaceflight-associated neuro-ocular syndrome because cerebral ventricular volume increases both in the supine position and in microgravity environments. We thought this is important because previous work (references 27-29) focused on sagittal sinus venous volume. Because CSF is privileged and independent of venous drainage, we thought this might be helpful to researchers in that field.

Reviewer #2: Very interesting and well written report describing the vasculature draining the CSF.

1. Please specify which lymphatic biomarkers were assessed to verify that the CSF vessels were not lymphatic vessels. Were labeled lymphatic vessels observed in the same histologic slides containing the CSF vessels? Including such an image could provide more evidence that these are indeed not lymphatic vessels. **RESPONSE:** Confocal laser imaging of LYVE-1 was included to show that it was negative for the biopsy of the cervical CSF canalicular system. Podoplanin (D2-40) was also negative but was not included for brevity. Negative CD105 was shown as our vascular endothelial marker. We included an image of a positive vimentin slide since this may be a consistent biomarker for CSF channels in the dura and in human peripheral nerve. The paper was re-written to emphasize the gross anatomy and to leave a more detailed molecular characterization for another project.
2. Figure 4: you have two types of arrows in figure (long and short) but only refer to arrows in the legend, please specify in the legend which arrows are being referenced. **RESPONSE**: Fig 4 was re-labelled with only one arrow and now appears as Figs 3a and 3b.
3. While indocyanine green fluorescence imaging is technically an 'infrared' technique, I recommend updating the text to refer to it as a 'near-infrared' technique to avoid potential confusion with thermal imaging which is often referred to as infrared imaging. **RESPONSE**: Thank you for pointing this out, we mislabeled the image. The near-infrared description was for another image (not used) and this was CT imaging after injection of iohexol (Omnipaque^TM^) dye. The Figure Legends for Figs 5a and 5b were corrected.

Thank you for reviewing our manuscript and for your criticisms and suggestions.
Sincerely,

Joel Pessa MD and Ronald Hoxworth MD MBA
