# Supplementary material for: Identification of a novel path for cerebrospinal fluid (CSF) drainage of the human brain
Source: PLoS One. 2023 May 4;18(5):e0285269. doi: 10.1371/journal.pone.0285269 (PMC10159342; doi:10.1371/journal.pone.0285269)
Supplement: Attachment — Submitted filename: Response to Reviewers.doc [file pone.0285269.s002.doc]

Response to Reviewers

**Additional Editor Comments**
The authors have responded satisfactorily to the initial reviewers' feedback. However, one of the reviewers has a few minor corrections which the authors need to address.

**Response**: the changes requested by Reviewer #1 have been made and inserted into lines 243-245, and into lines 249-252. The references have been verified. Thank you for reviewing our manuscript.

**Reviewer #1**

1. The data showing increased ventricular volume are from imaging done AFTER spaceflights. The changes in ventricular volume could have occurred either in space or during the re-adaptation to one-G. With that in mind change:
"SANS is thought to be related to dysregulation of CSF flow (27-29). Is SANS related to decreased drainage in the CSF canalicular system?"

to

”SANS may be related to dysregulation of CSF flow (27-29) and any possible role of the CSF canalicular system in SANS could be investigated.”

**Response**: Lines 243-245 have been changed to **“SANS may be related to dysregulation of CSF flow (27-29) and any possible role of the CSF canalicular system in SANS could be investigated.”**

2. …and change: "The reverse is noted during spaceflight, where cerebral ventricular volume is increased in low-gravity environments (31). The increased thickness of the optic nerve sheath is explained by stasis of flow in CSF channels found in the optic nerve dura (Fig 11a) (32)."

to

”The reverse has been noted after spaceflight. Cerebral ventricular volume may be increased in low-gravity environments or after return to 1 G (31). Some authors propose the increased optic nerve sheath thickness is explained by stasis of flow in CSF channels found in the optic nerve dura (Fig 11a) (32).”

**Response:** Lines 249-252 have been changed to **“The reverse has been noted after spaceflight. Cerebral ventricular volume may be increased in low-gravity environments or after return to 1 G (31). Some authors propose the increased optic nerve sheath thickness is explained by stasis of flow in CSF channels found in the optic nerve dura (Fig 11a) (32).”**

Thank you for reviewing our manuscript and for pointing out that changes could occur during re-adaptation to 1G.

Reviewer #2: (No Response)

**Response**: Thank you for reviewing our manuscript.
